# 3-Methyl-6-trichloro­methyl-1,2,4-triazolo[3,4-*b*][1,3,4]thia­diazole

**DOI:** 10.1107/S1600536811012748

**Published:** 2011-04-13

**Authors:** Wei-min Jia, Zhi-jian Wang, Xiao-yu Jia, Jing-jing Zhang, Wei Wang

**Affiliations:** a School of Perfume and Aroma Technology, Shanghai Istitute of Technology, Shanghai 200235, People’s Republic of China; b School of Chemical Engineering, University of Science and Technology LiaoNing, Anshan 114051, People’s Republic of China

## Abstract

In the crystal structure of the title compound, C_5_H_3_Cl_3_N_4_S, two mol­ecules related by a centre of symmetry demonstrate extremely short inter­molecular S⋯N contacts of 2.783 (2) Å. The crystal packing also exhibits π–π inter­actions indicated by a short distance of 3.340 (1) Å between the centroids of the triazole rings of neighbouring mol­ecules.

## Related literature

For the anti­microbial and anti-inflammatory activity of 1,2,4-triazole and 1,3,4-thio­diazole derivatives, see: Karabasanagouda *et al.* (2007 ▶); Mathew *et al.* (2007 ▶); For related structures, see: Du *et al.* (2008 ▶); Khan *et al.* (2009 ▶); Haugwitz *et al.* (1977 ▶). 
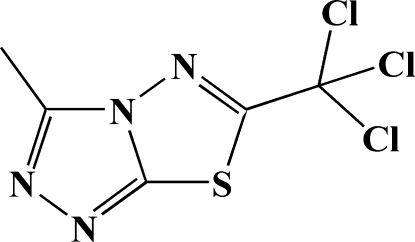


## Experimental

### 

#### Crystal data

C_5_H_3_Cl_3_N_4_S
*M* *_r_* = 257.52
Monoclinic, 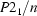

*a* = 5.8732 (12) Å
*b* = 9.4164 (19) Å
*c* = 16.750 (3) Å
β = 91.82 (3)°
*V* = 925.9 (3) Å^3^
*Z* = 4
Mo *K*α radiation
μ = 1.17 mm^−1^
*T* = 153 K
0.30 × 0.20 × 0.10 mm

#### Data collection

Rigaku Saturn CCD area-detector diffractometer
Absorption correction: multi-scan (*CrystalClear*; Rigaku/MSC, 2005 ▶) *T*_min_ = 0.721, *T*_max_ = 0.892
9841 measured reflections
2196 independent reflections
1934 reflections with *I* > 2σ(*I*)
*R*_int_ = 0.038

#### Refinement

*R*[*F* ^2^ > 2σ(*F* ^2^)] = 0.029
*wR*(*F* ^2^) = 0.113
*S* = 1.24
2196 reflections
120 parameters
H-atom parameters constrained
Δρ_max_ = 0.49 e Å^−3^
Δρ_min_ = −0.44 e Å^−3^

### 

Data collection: *CrystalClear* (Rigaku/MSC, 2005 ▶); cell refinement: *CrystalClear*; data reduction: *CrystalClear*; program(s) used to solve structure: *SHELXS97* (Sheldrick, 2008 ▶); program(s) used to refine structure: *SHELXL97* (Sheldrick, 2008 ▶); molecular graphics: *SHELXTL* (Sheldrick, 2008 ▶); software used to prepare material for publication: *SHELXTL*.

## Supplementary Material

Crystal structure: contains datablocks global, I. DOI: 10.1107/S1600536811012748/cv5070sup1.cif

Structure factors: contains datablocks I. DOI: 10.1107/S1600536811012748/cv5070Isup2.hkl

Additional supplementary materials:  crystallographic information; 3D view; checkCIF report

## Figures and Tables

**Fig. 1.**
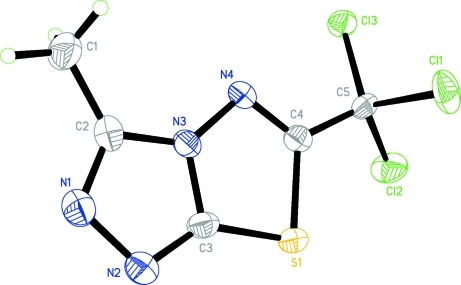
View of the molecule of (I) showing the atom-labelling scheme. Displacement ellipsoids are drawn at the 60% probability level.

## supplementary crystallographic information

### Comment

1,2,4-Triazole and 1,3,4-thiodiazole derivatives demonstrate various activities such as antimicrobial (Karabasanagouda *et al.*, 2007) and anti-inflammatory (Mathew *et al.*, 2007) activities. Herewith we report the synthesis and crystal structure of the title compound (I), a new derivative from the aforementioned family.

In (I) (Fig. 1), all bond lengths and angles are normal and correspond to those observed in the related structures (Du *et al.*, 2008; Khan *et al.*, 2009). The triazolothiadiazole ring system is essentially planar with an r.m.s derivation of 0.0087 (2)Å and maximum deviation of 0.0037 (2)Å for atom C2. In the crystal structure, π-π interactions (Table 1) consolidate the crystal packing, which exhibits short intermolecular S···N contacts of 2.783 (2) Å observed eralier in the related structure (Haugwitz *et al.*, 1977).

### Experimental

The title compound was synthesized by the reaction of 4-amino-3-methyl-4*H*-1,2,4-triazole-5-thiol (2.0 mmol) and trichloroacetic acid (2.0 mmol) in phosphoryl trichloride for 24 h. Crystals of (I) suitable for single-crystal X-ray analysis were grown by slow evaporation of a solution in chloroform-ethanol (1:1).

### Refinement

H atoms were positioned geometrically (C—H = 0.98 Å) and refined as riding, with *U*_iso_(H) = 1.5*U*_eq_(parent).

### Crystal data

**Table:**

C_5_H_3_Cl_3_N_4_S	*F*(000) = 512
*M**_r_* = 257.52	*D*_x_ = 1.847 Mg m^−^^3^
Monoclinic, *P*2_1_/*n*	Mo *K*α radiation, λ = 0.71073 Å
Hall symbol: -P 2yn	Cell parameters from 2918 reflections
*a* = 5.8732 (12) Å	θ = 3.3–27.9°
*b* = 9.4164 (19) Å	µ = 1.17 mm^−^^1^
*c* = 16.750 (3) Å	*T* = 153 K
β = 91.82 (3)°	Prism, colorless
*V* = 925.9 (3) Å^3^	0.30 × 0.20 × 0.10 mm
*Z* = 4	

### Data collection

**Table:**

Rigaku Saturn CCD area-detector diffractometer	2196 independent reflections
Radiation source: rotating anode	1934 reflections with *I* > 2σ(*I*)
multilayer	*R*_int_ = 0.038
Detector resolution: 7.31 pixels mm^-1^	θ_max_ = 27.9°, θ_min_ = 2.4°
φ and ω scans	*h* = −7→7
Absorption correction: multi-scan (*CrystalClear*; Rigaku/MSC, 2005)	*k* = −10→12
*T*_min_ = 0.721, *T*_max_ = 0.892	*l* = −22→22
9841 measured reflections	

### Refinement

**Table:**

Refinement on *F*^2^	Secondary atom site location: difference Fourier map
Least-squares matrix: full	Hydrogen site location: inferred from neighbouring sites
*R*[*F*^2^ > 2σ(*F*^2^)] = 0.029	H-atom parameters constrained
*wR*(*F*^2^) = 0.113	*w* = 1/[σ^2^(*F*_o_^2^) + (0.0693*P*)^2^] where *P* = (*F*_o_^2^ + 2*F*_c_^2^)/3
*S* = 1.24	(Δ/σ)_max_ < 0.001
2196 reflections	Δρ_max_ = 0.49 e Å^−^^3^
120 parameters	Δρ_min_ = −0.44 e Å^−^^3^
0 restraints	Extinction correction: *SHELXL97* (Sheldrick, 2008), Fc^*^=kFc[1+0.001xFc^2^λ^3^/sin(2θ)]^-1/4^
Primary atom site location: structure-invariant direct methods	Extinction coefficient: 0.071 (6)

### Special details

**Table:**

Geometry. All e.s.d.'s (except the e.s.d. in the dihedral angle between two l.s. planes) are estimated using the full covariance matrix. The cell e.s.d.'s are taken into account individually in the estimation of e.s.d.'s in distances, angles and torsion angles; correlations between e.s.d.'s in cell parameters are only used when they are defined by crystal symmetry. An approximate (isotropic) treatment of cell e.s.d.'s is used for estimating e.s.d.'s involving l.s. planes.
Refinement. Refinement of *F*^2^ against ALL reflections. The weighted *R*-factor *wR* and goodness of fit *S* are based on *F*^2^, conventional *R*-factors *R* are based on *F*, with *F* set to zero for negative *F*^2^. The threshold expression of *F*^2^ > σ(*F*^2^) is used only for calculating *R*-factors(gt) *etc*. and is not relevant to the choice of reflections for refinement. *R*-factors based on *F*^2^ are statistically about twice as large as those based on *F*, and *R*- factors based on ALL data will be even larger.

### Fractional atomic coordinates and isotropic or equivalent isotropic displacement parameters (Å^2^)

**Table:**

	*x*	*y*	*z*	*U*_iso_*/*U*_eq_	
S1	0.13777 (9)	0.52705 (5)	0.13657 (3)	0.01622 (19)	
Cl1	0.55694 (10)	0.67616 (5)	0.27560 (3)	0.0242 (2)	
Cl2	0.15047 (9)	0.53079 (6)	0.32341 (3)	0.02410 (19)	
Cl3	0.58496 (8)	0.38759 (5)	0.33267 (3)	0.01648 (18)	
N1	0.3533 (3)	0.30847 (19)	−0.04370 (11)	0.0193 (4)	
N2	0.1853 (3)	0.40411 (19)	−0.01768 (11)	0.0191 (4)	
N3	0.4320 (3)	0.35972 (16)	0.08095 (10)	0.0134 (4)	
N4	0.5132 (3)	0.37661 (17)	0.15796 (10)	0.0133 (4)	
C1	0.7018 (4)	0.1903 (2)	0.01481 (13)	0.0223 (5)	
H1A	0.7271	0.1594	−0.0401	0.033*	
H1B	0.6776	0.1070	0.0486	0.033*	
H1C	0.8353	0.2430	0.0352	0.033*	
C2	0.4981 (3)	0.2833 (2)	0.01591 (12)	0.0158 (4)	
C3	0.2385 (3)	0.4306 (2)	0.05699 (12)	0.0151 (4)	
C4	0.3744 (3)	0.46089 (19)	0.19257 (12)	0.0135 (4)	
C5	0.4165 (3)	0.5096 (2)	0.27709 (11)	0.0136 (4)	

### Atomic displacement parameters (Å^2^)

**Table:**

	*U*^11^	*U*^22^	*U*^33^	*U*^12^	*U*^13^	*U*^23^
S1	0.0153 (3)	0.0166 (3)	0.0165 (3)	0.00419 (18)	−0.0027 (2)	−0.00059 (17)
Cl1	0.0359 (4)	0.0136 (3)	0.0227 (3)	−0.0086 (2)	−0.0055 (2)	0.00025 (18)
Cl2	0.0186 (3)	0.0337 (4)	0.0202 (3)	0.0075 (2)	0.0050 (2)	−0.0021 (2)
Cl3	0.0186 (3)	0.0164 (3)	0.0143 (3)	0.00304 (18)	−0.00199 (19)	0.00178 (16)
N1	0.0204 (9)	0.0206 (9)	0.0169 (9)	−0.0011 (7)	−0.0001 (7)	−0.0020 (7)
N2	0.0201 (9)	0.0214 (9)	0.0157 (9)	0.0021 (7)	−0.0023 (7)	−0.0011 (7)
N3	0.0142 (8)	0.0127 (8)	0.0133 (8)	−0.0010 (6)	−0.0022 (6)	0.0002 (6)
N4	0.0132 (8)	0.0145 (8)	0.0122 (8)	−0.0015 (6)	−0.0015 (6)	0.0002 (6)
C1	0.0240 (12)	0.0254 (11)	0.0176 (10)	0.0059 (9)	0.0015 (8)	−0.0031 (8)
C2	0.0180 (10)	0.0155 (9)	0.0142 (9)	−0.0029 (8)	0.0017 (7)	−0.0013 (7)
C3	0.0133 (10)	0.0135 (9)	0.0184 (10)	0.0013 (8)	−0.0018 (8)	0.0022 (7)
C4	0.0145 (10)	0.0121 (9)	0.0136 (9)	−0.0009 (7)	−0.0007 (7)	0.0027 (7)
C5	0.0138 (9)	0.0110 (8)	0.0162 (10)	0.0023 (7)	0.0008 (7)	0.0009 (7)

### Geometric parameters (Å, °)

**Table:**

S1—C3	1.732 (2)	N3—N4	1.370 (2)
S1—C4	1.765 (2)	N3—C2	1.372 (3)
Cl1—C5	1.772 (2)	N4—C4	1.289 (3)
Cl2—C5	1.778 (2)	C1—C2	1.483 (3)
Cl3—C5	1.763 (2)	C1—H1A	0.9800
N1—C2	1.313 (3)	C1—H1B	0.9800
N1—N2	1.415 (2)	C1—H1C	0.9800
N2—C3	1.304 (3)	C4—C5	1.501 (3)
N3—C3	1.367 (3)		
			
Cg1···Cg1^i^	3.340 (1)	Cg1···Cg2^i^	3.682 (1)
			
C3—S1—C4	86.65 (9)	N1—C2—C1	126.9 (2)
C2—N1—N2	108.78 (17)	N3—C2—C1	124.63 (18)
C3—N2—N1	105.63 (17)	N2—C3—N3	111.08 (18)
C3—N3—N4	118.75 (16)	N2—C3—S1	139.59 (16)
C3—N3—C2	106.06 (17)	N3—C3—S1	109.33 (14)
N4—N3—C2	135.19 (17)	N4—C4—C5	121.67 (18)
C4—N4—N3	106.78 (16)	N4—C4—S1	118.48 (15)
C2—C1—H1A	109.5	C5—C4—S1	119.77 (15)
C2—C1—H1B	109.5	C4—C5—Cl3	111.82 (14)
H1A—C1—H1B	109.5	C4—C5—Cl1	108.66 (13)
C2—C1—H1C	109.5	Cl3—C5—Cl1	109.30 (11)
H1A—C1—H1C	109.5	C4—C5—Cl2	108.97 (14)
H1B—C1—H1C	109.5	Cl3—C5—Cl2	109.20 (10)
N1—C2—N3	108.44 (17)	Cl1—C5—Cl2	108.84 (10)
			
C2—N1—N2—C3	−0.4 (2)	C2—N3—C3—S1	178.46 (13)
C3—N3—N4—C4	0.9 (2)	C4—S1—C3—N2	179.8 (3)
C2—N3—N4—C4	−178.6 (2)	C4—S1—C3—N3	0.80 (14)
N2—N1—C2—N3	−0.2 (2)	N3—N4—C4—C5	−176.81 (17)
N2—N1—C2—C1	−179.41 (19)	N3—N4—C4—S1	−0.2 (2)
C3—N3—C2—N1	0.6 (2)	C3—S1—C4—N4	−0.36 (16)
N4—N3—C2—N1	−179.8 (2)	C3—S1—C4—C5	176.31 (16)
C3—N3—C2—C1	179.86 (19)	N4—C4—C5—Cl3	−25.8 (2)
N4—N3—C2—C1	−0.6 (3)	S1—C4—C5—Cl3	157.64 (11)
N1—N2—C3—N3	0.7 (2)	N4—C4—C5—Cl1	94.93 (19)
N1—N2—C3—S1	−178.24 (19)	S1—C4—C5—Cl1	−81.63 (16)
N4—N3—C3—N2	179.50 (16)	N4—C4—C5—Cl2	−146.61 (16)
C2—N3—C3—N2	−0.8 (2)	S1—C4—C5—Cl2	36.83 (18)
N4—N3—C3—S1	−1.2 (2)		

Symmetry codes: (i) −*x*+1, −*y*+1, −*z*.
